# Combating Diagnostic Delay of Endometriosis in Adolescents via Educational Awareness: A Systematic Review

**DOI:** 10.7759/cureus.15143

**Published:** 2021-05-20

**Authors:** Cassandra N Simpson, Christine M Lomiguen, Justin Chin

**Affiliations:** 1 Obstetrics and Gynecology, Lake Erie College of Osteopathic Medicine, Erie, USA; 2 Pathology, Lake Erie College of Osteopathic Medicine, Erie, USA; 3 Family Medicine, Millcreek Community Hospital, Erie, USA; 4 Medical Education, Lake Erie College of Osteopathic Medicine, Erie, USA; 5 Family Medicine, LifeLong Medical Care, Richmond, USA

**Keywords:** endometriosis, adolescents, pelvic pain, general gynecology, medical education

## Abstract

Endometriosis occurs in approximately 10% of adult women worldwide; however, it is commonly under- or misdiagnosed in adolescents. The purpose of this study was to analyze existing scientific literature for reasons for diagnostic delay of endometriosis and to determine how education regarding endometriosis could be improved. An integrative review was conducted based on articles published between December 1980 and December 2020. Suitable articles were identified from the MEDLINE/PubMed databases, using relevant terms. Eligible studies included discussion on potential causes of diagnostic delay of endometriosis in the adolescent population. Data were extracted from eligible publications and qualitative synthesis was used. The 27 articles included in the study revealed several primary reasons for the delay, such as a physician and/or patient knowledge gap, normalization by physician and patient, lack of research, and physician resistance. Strategies to lessen diagnostic delay of endometriosis in adolescents must include integrated actions by educators and healthcare providers to improve health literacy and awareness of common causes of pelvic pain in this age group.

## Introduction and background

Endometriosis is defined as endometrial-like glands or stroma outside of the uterine cavity that leads to an estrogen-dependent chronic inflammatory state. This endometrial-like tissue is classically found on the ovaries, uterine ligaments, and rectovaginal septum [1]. Endometriosis is present in approximately 10% of adult women worldwide, but the prevalence in adolescent populations has been difficult to quantify; estimates have varied among studies [2]. Endometriosis is the leading cause of chronic pelvic pain and most often presents in adolescents with dysmenorrhea, or acyclic pelvic pain. Endometriosis and its attributable symptoms can greatly affect an adolescent’s life, causing absenteeism in school, decreased socialization, and sexual impairment [3]. Characteristics associated with increased risk of endometriosis involve prolonged exposure to estrogen, such as shorter menstrual cycles, early menarche, or late menopause [4], as well as a family history of endometriosis, and Mullerian anomalies [5].

**Figure 1:**
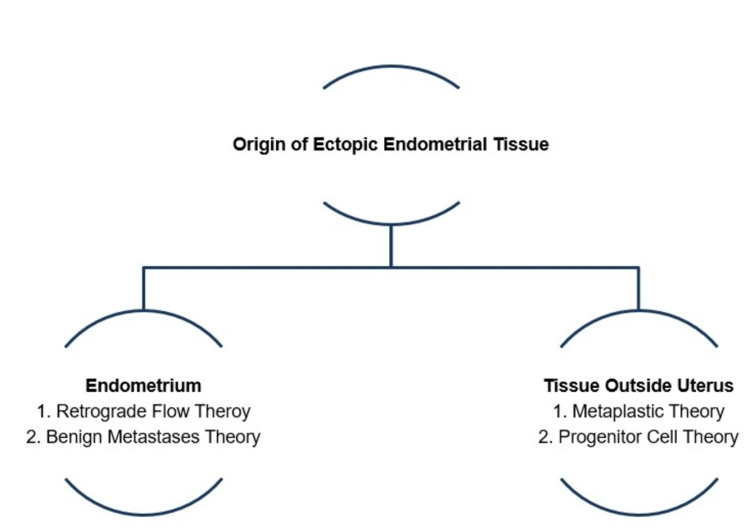
Supported Theories of the Pathogenesis of Endometriosis

Adolescents may be affected by endometriosis, or early-onset endometriosis, contingent upon the timeline of symptoms and menarche. A unanimous theory regarding the pathogenesis of endometriosis has yet to be elucidated; however, widely accepted theories fall into one of two categories in relation to the origin of ectopic endometrial tissue (Figure 1). One category proposes ectopic endometrial tissue originates in the individual’s endometrium. The major theory that falls into this category is the retrograde flow theory, in which it is assumed that retrograde flow of endometrial tissue during menstruation leads to ectopic implantation. Similarly, the benign metastases theory suggests the spread of endometrial tissue can occur through lymphatic and hematogenous channels. Contrarily, the other proposed category suggests tissues outside of the uterus give rise to endometrial-like glands and stroma. For example, the metaplastic theory favors the idea of coelomic epithelium and mesonephric remnants undergoing transformation into ectopic endometrial tissue. Lastly, the progenitor cell theory illustrates the possibility of progenitor cells directly from bone marrow differentiating into ectopic endometrial tissue [1].

Furthermore, recent evidence suggests the cause of early-onset endometriosis, defined as presenting prior to menarche, is neonatal uterine bleeding [2]. This process involves the implantation of endometrial progenitor cells into the pelvic cavity. Bleeding can occur in the postnatal period secondary to maternal progesterone withdrawal. These naive endometrial cells can be seeded in the peritoneum, and then become activated during thelarche [5]. Thus, individuals with early-onset endometriosis may experience symptoms prior to menarche [2].

The aim of this review was to assess the major causes of diagnostic delay of endometriosis, specifically in adolescents. Addressing major causes for delay and recognizing how these factors are potentially affected by increased digital education have the potential to mitigate further setbacks in the diagnostic timeline. Moreover, suggestions are included for incorporating information regarding pelvic pain syndromes, such as endometriosis, in formal educational settings for premenarcheal women.

## Review

Methods

**Figure 2:**
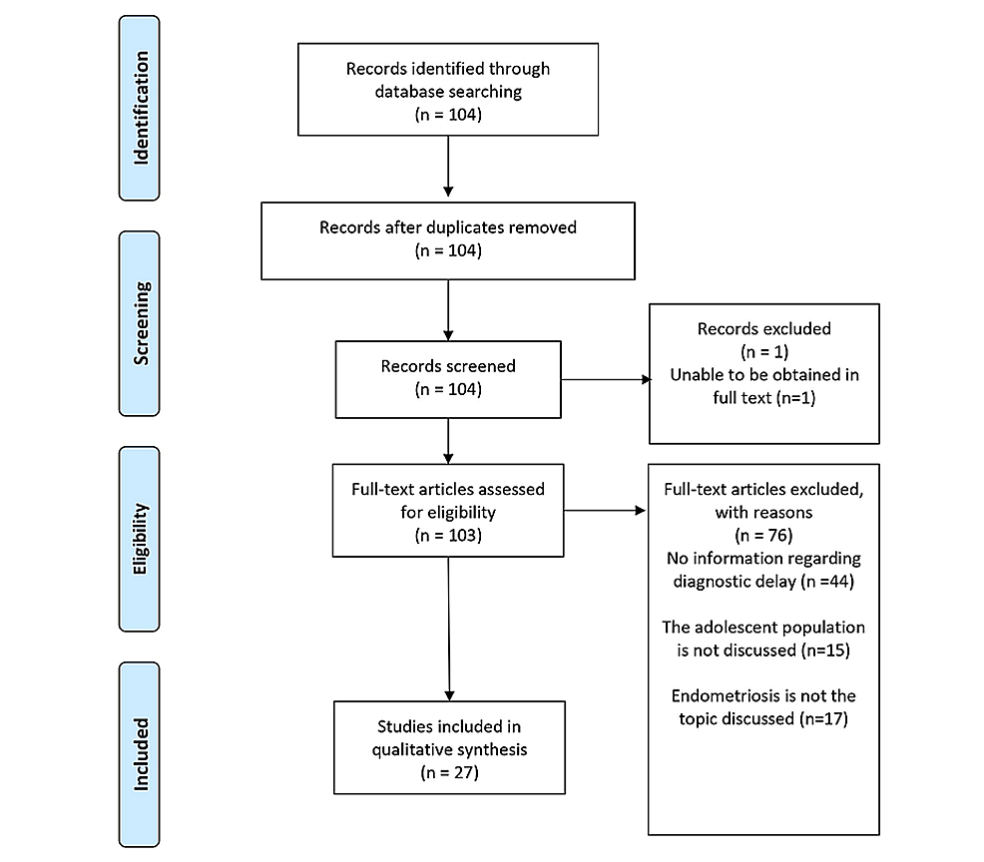
PRISMA Flow Chart Indicating the Inclusion of Manuscripts Regarding the Diagnostic Delay of Endometriosis

A search was conducted of the National Library of Medicine’s MEDLINE/PubMed databases with the intent of finding all articles published in the English language from 1980 to 2020 with “endometriosis” in conjunction with “diagnostic delay” and “adolescent” or “adolescence”. Articles identified by this search strategy were read in full and analyzed for relevance. Additionally, corresponding bibliographic reference sections were reviewed for additional studies not found by the previous method. All articles were initially accessed between August 2020 and November 2020. A total of one hundred four (104) articles were reviewed and retrieved independently by the first author. In order to be included, a discussion of potential causes of diagnostic delay of endometriosis in the adolescent population must have been present in the publication. One article was not able to be obtained in full text and was thus excluded. Forty-four (44) articles did not possess relevant information regarding diagnostic delay of endometriosis and were thus excluded. Fifteen (15) articles were excluded due to a lack of information regarding the adolescent population. Lastly, relevant aspects of endometriosis were not discussed in seventeen (17) articles that were ultimately excluded. A total of twenty-seven (27) manuscripts met the criteria and were used in this review (Figure 2). The quality of studies was assessed using National Institutes of Health (NIH) quality assessment tools [6]. Each study was classified as low risk (≥7), moderate risk (5-6), or high risk of bias (≤4).

Results

A total of twenty-seven (27) articles [5,7-32] were selected to illustrate the different reasons for the diagnostic delay of endometriosis in adolescents. From these 27 articles, thirty-two (32) reasons for the diagnostic delay were extrapolated and classified into one of six categories. The analysis and categorization of the reasons for delay are conveyed in Table 1.

**Table 1:** Summary of Reasons for Diagnostic Delay of Endometriosis in Adolescents

Author/Year	Publication Bias	Type of Study	Participants	Age	Cause of Diagnostic Delay	Category
Gubbels et al., 2020 [5]	4	Review	NA	10-19	Misunderstanding of the complex disease process; differences in presentation between adolescents and adults	Physician knowledge gap
Suvitie et al., 2016 [7]	8	Cross-Sectional Study	1103	15-19	Adolescents waited 3 times longer than adults to seek treatment for their symptoms	Normalization by Patient
Sachedina et al., 2020 [8]	2	Review	NA	10-19	Normalization of dysmenorrhea by a physician	Normalization by Physician
Pirtea et al., 2020 [9]	1	Review	NA	10-19	Normalization of dysmenorrhea by a physician	Normalization by Physician
Pirtea et al., 2020 [9]	1	Review	NA	10-19	Physicians resistant to conduct the laparoscopic procedure in adolescents	Physician resistance
Sieberg et al., 2020 [10]	2	Review	NA	10-19	Lack of endometriosis research specifically for adolescent presentation	Lack of Research
Sieberg et al., 2020 [10]	2	Review	NA	10-19	Lesions in adolescents are usually clear, white, and/ or yellow-brown. Lesions in adults are usually black or blue	Physician knowledge gap
Dun et al., 2015 [11]	9	Retrospective Case Series	288	10-19	Presence of atypical lesions	Physician knowledge gap
Dun et al., 2015 [11]	9	Retrospective Case Series	288	10-19	Adolescents may experience different symptoms, such as vague abdominal discomfort, GI distress, and genitourinary symptoms	Physician knowledge gap
Staal et al., 2016 [12]	7	Retrospective Cohort Study	93	>18	Infertility, a symptom not readily recognized in adolescents, may result in a faster diagnosis in adults.	Physician knowledge gap
Shadbolt et al., 2013 [13]	7	Survey	131	16-25	Lack of patient knowledge and understanding of the disease	Patient Knowledge Gap
Youngster et al., 2013 [14]	7	Cross-Sectional Study	295	<18	Unique presentation in adolescents leads to high rates of misdiagnosis	Physician knowledge gap
Brosens et al., 2013 [15]	5	Systematic Review	437	10-21	Dysmenorrhea and acyclic menstrual pain are common complaints that often do not elicit a further investigation	Normalization by Physician
Brosens et al., 2013 [15]	5	Systematic Review	437	10-12	A differential diagnosis that is focused more on the gastrointestinal system due to symptoms such as abdominal pain and GI dysfunction	Physician knowledge gap
Santos et al., 2012 [16]	4	Retrospective Analysis	262	<20	Resistant to consider this diagnosis in younger patients	Physician resistance
Pugsley et al., 2007 [17]	8	Retrospective Analysis	101	>16	Ultrasound is commonly ordered, but only helpful in diagnosing about 10% of cases	Physician knowledge gap
Chapron, 2002 [18]	8	Retrospective Analysis	160	19-51	Speculum exam allows endometriotic lesions to be viewed in only 14.4% of patients	Physician knowledge gap
Seear, 2009 [19]	1	Qualitative Survey	20	24-55	Normalization of menstrual pain	Normalization by Physician
Seear, 2009 [19]	1	Qualitative Survey	20	24-55	Women are often reluctant to disclose menstrual irregularities	Normalization by Patient
Troyer, 2007 [20]	7	Case Study	1	25	Adolescents may experience different symptoms, such as low back and buttock pain, for which endometriosis is not part of the differential diagnosis.	Physician knowledge gap
Ballweg, 2003 [21]	1	Opinion Piece	NA	NA	Girls take an average of 4.67 years to report symptoms to their doctor	Normalization by patient
ACOG, 2005 [22]	1	Committee Opinion	NA	10-19	Imaging studies, such as ultrasound, and serum markers, such as CA 125 can be used to diagnose endometriosis in adults, but are rarely useful in diagnosing adolescents	Physician knowledge gap
Laufer et al., 2003 [23]	3	Review	NA	10-19	Adolescents may have subtle signs of endometriosis on laparoscopy	Physician knowledge gap
Garcia et al., 2003 [24]	7	Case Study	1	18	Delayed diagnosis of underlying congenital anomaly that leads to endometriosis as a complication	Physician knowledge gap
De Sanctis et al., 2018 [25]	3	Review	NA	<25	Lack of standard methods for assessing the severity of symptoms in adolescents	Lack of Research
De Sancis et al., 2018 [25]	3	Review	NA	<25	Subtle, atypical lesions that are red or clear instead of brown are more common in adolescents	Physician knowledge gap
Parasar et al., 2013 [26]	1	Review	NA	NA	High cost of diagnosis and treatment in adolescents	Physician Resistance
Gordts et al., 2015 [27]	2	Review	NA	NA	Mild or complete lack of pelvic pain symptoms	Physician knowledge gap
Fong et al., 2017 [28]	9	Retrospective Chart Review	45	14-25	Cultural reluctance to visit a gynecologist at a young age	Patient knowledge gap
Fong et al., 2017 [28]	9	Retrospective Chart Review	45	14-25	Symptoms are different than adult presentation; may include dyschezia, dysuria, urgency, hematuria	Physician knowledge gap
DiVasta et al., 2018 [29]	7	Cross-Sectional Study	402	<18	Nausea in conjunction with pelvic pain is a major symptom in adolescents	Physician knowledge gap
Shim et al., 2020 [30]	2	Review	NA	10-19	Infrequent presentation of endometrioma or infertility, the major symptoms in adults	Physician knowledge gap
Galczyznski et al., 2019 [31]	5	Review	NA	11-19	Adolescents tend to wait longer to seek professional help	Normalization by Patient
Martire et al., 2020 [32]	12	Retrospective Observational Study	270	12-20	Misdiagnosis	Physician knowledge gap

Six (6) reasons for the diagnostic delay of endometriosis in adolescents were identified through this literature review: four (4) physician-related reasons (knowledge gap, normalization, lack of research, and resistance) and two (2) patient-related reasons (knowledge gap and normalization), with physician knowledge gap as the most identified reason. The number of articles in each category is conveyed in Table 2. 

**Table 2:** Summary of Articles Categorized into Patient- and Physician-Related Causes of Diagnostic Delay

Reason for Diagnostic Delay		Number of Cited Articles
Physician-related	Physician Knowledge Gap	19
	Normalization by Physician	4
	Lack of Research	2
	Physician Resistance	3
Patient-related	Patient Knowledge Gap	2
	Patient Normalization	4

Each study was individually evaluated for publication using the NIH quality assessment scales. Using these scales, studies were classified as low risk (>7), medium risk (5-6), or high risk of bias (<4). The overall risk of publication bias across all studies used was 4.92, indicating a moderate risk of bias. All review articles lacked independent quality ratings of studies that were used. Similarly, all lacked an overall publication bias, ultimately leading to lower scores on the NIH quality assessment scale. Review studies comprised the largest cohort of studies, and therefore remain the greatest risk for bias.

Discussion

Patient-Related Causes of Diagnostic Delay

Among the categories determined for causes of diagnostic delay, two placed the cause of delay on the patient seeking treatment. Normalization of dysmenorrhea and other menstrual pain symptoms caused adolescent women to wait three times longer to seek medical treatment than adults with the same symptoms [7].

Similarly, a patient’s own knowledge gap may lead to delayed diagnosis. Historically, healthcare providers were the primary sources for learning about endometriosis; however, the rise of the use of the internet has allowed for a more individualized approach to symptom recognition [13]. Comprehensive sexual education through the education system has been shown to advance awareness in both traditional sexual health, such as sexually transmitted infections (STIs) and pregnancy, as well as adjacent topics, such as dating and intimate partner violence, appreciation of sexual diversity, and development of healthy relationships [33]. There lacks, however, a formal curriculum in the United States for adolescent awareness of endometriosis and its symptoms. A pilot program in New Zealand has suggested that an endometriosis-focused curriculum increases awareness of the condition in adolescent students with subsequent earlier presentations to appropriate healthcare providers for clinical assessment, diagnosis, and treatment [34]. However, the expansion of digital education has produced dissatisfaction with learning due to poor internet connection, methods of presentation, and level of participation in class activities. This could ultimately lead to a decrease in adolescent knowledge of health conditions [35].

Physician-Related Causes of Diagnostic Delay

Four categories placed the cause of delay on the physician. Providers may lack proper insight on the presentation of endometriosis in adolescents compared to adults. Gaps in education can ultimately lead to gaps in knowledge and lack of comprehension, resulting in the inability to diagnose or treat certain diseases. Physicians were also shown to have reluctance in including endometriosis as part of their differential diagnosis when patients presented with a chief complaint of severe back pain or gastrointestinal symptoms rather than the standard menstrual symptoms [10,11]. While these are presenting symptoms of an adolescent with endometriosis, many health care providers fail to recognize these symptoms as such. Similarly, patients who reported dyspareunia and dysmenorrhea were found to have a longer delay in diagnosis, than those who denied those symptoms [16]. A lack of research specifically for adolescent endometriosis exists, and ultimately may be ignored as a potential diagnosis. Common symptoms, such as dysmenorrhea, often do not elicit further evaluation by a physician, indicating that symptoms of menstrual pain have been normalized by physicians [15].

Moreover, it is common for physicians to attempt to diagnose endometriosis using an ultrasound machine, or speculum exam, but these methods have not been shown to successfully diagnose adolescents [17]. Laparoscopy, which is a necessary procedure to achieve a definitive diagnosis, is only recommended after the patient’s symptoms have failed to subside after medical treatment with non-steroidal anti-inflammatory drugs (NSAIDs) and estrogen/progestin or progestin-only therapy [30]. Physicians may be resistant to perform laparoscopic procedures on adolescents due to the invasiveness and cost of the procedure. However, it is through laparoscopy that the most accurate diagnosis may be made [30].

Endometriosis Education

The CDC outlines current guidelines for formulating a sexual health curriculum utilizing eight Healthy Behavior Outcomes (HBO) in grades pre-kindergarten through 12th grade (Table 3). HBO number eight includes promoting the use of health services to promote sexual health [36]. The result of this review suggests that this category should be broadened to include information regarding menstrual pain and pelvic pain syndromes that could ultimately affect a young woman’s life. Adolescents often are unprepared for menarche when their education system fails to teach them about it. These patients are therefore not equipped to properly handle symptoms that may accompany menarche and are unlikely to recognize if symptoms are abnormal. Furthermore, the expansion of digital education has produced dissatisfaction with learning due to poor internet connection, methods of presentation, and level of participation in class activities. This could ultimately lead to a decrease in adolescent knowledge of health conditions [35]. The appropriate use of health services to promote sexual health should encompass the normal and abnormal changes that may present alongside sexual development.

**Table 3:** Center for Disease Control Healthy Behavior Outcomes for Students in Pre-Kindergarten through 12th Grade

Healthy Behavior Outcomes (HBO)
HBO 1: Establish and Maintain healthy relationships
HBO 2: Be sexually abstinent
HBO 3: Engage in behaviors that prevent or reduce sexually transmitted disease, including HIV infection
HBO 4: Engage in behaviors that prevent or reduce unintended pregnancy
HBO 5: Avoid pressuring others to engage in sexual behaviors
HBO 6: Support others to avoid or reduce sexual risk behaviors
HBO 7: Treat others with courtesy and respect without regard to their sexuality
HBO 8: Use appropriate health services to promote sexual health

## Conclusions

The information in this review is limited by the moderate risk of bias determined using the NIH quality assessment scale. Similarly, there is a risk of misclassification of reasons for the diagnostic delay. Future studies can suggest specific educational strategies to increase both physician, patient, and adolescent population knowledge regarding endometriosis to address the identified categories of diagnostic delay by lessening knowledge gaps, preventing normalization, decreasing resistance, and encouraging additional research.

Ultimately, the diagnostic delay of endometriosis highlights the need for increased health literacy, specifically the education of young females with respect to common pelvic pain syndromes. Gynecologists, as well as primary care providers and health educators, play a key role in mitigating the effect of endometriosis on an adolescent’s life, and therefore, must be aware of all possible presenting symptoms.
